# Biomimetic mineralization of electrospun elastin-like recombinamer nanofibers

**DOI:** 10.1038/s41598-026-58373-6

**Published:** 2026-06-22

**Authors:** Caixia Lan, Daniel Moreno, J. Carlos Rodríguez-Cabello, Lina Niu, Conrado Aparicio

**Affiliations:** 1 Minnesota Dental Research Center for Biomaterials and Biomechanics, School of Dentistry, University of Minnesota, Minneapolis, MN 55455 USA; 2 Bioinspired Oral Biomaterials and Interfaces (BOBI) Lab, Department of Materials Science and Engineering and Barcelona Research Centre in Multiscale Science and Engineering (CCEM), Barcelona East School of Engineering (EEBE), Universitat Politècnica de Catalunya -Barcelona Tech (UPC), Barcelona, 08034 Spain; 3 BIOFORGE Lab, University of Valladolid, CIBER-BBN, LaDIS, Valladolid, 47011 Spain; 4 State Key Laboratory of Oral & Maxillofacial Reconstruction and Regeneration, National Clinical Research Center for Oral Diseases, Shaanxi Key Laboratory of Stomatology, Department of Prosthodontics, School of Stomatology, The Fourth Military Medical University, Xi’an, 710032 Shaanxi China; 5 Catalan Institute for Research and Advanced Studies (ICREA), Barcelona, 08010 Spain; 6 Institute for Bioengineering of Catalonia (IBEC), C/. Baldiri Reixac 10-12, Barcelona, 08028 Spain

**Keywords:** Biomineralization, Electrospinning, Elastin-like recombinamers (ELR), Hard tissue regeneration, Biotechnology, Materials science

## Abstract

One of the key factors for hard tissue regeneration is to develop mineralized fibrous scaffolds. These scaffolds can provide a micro-environment resembling the structure and mechanical properties of the extracellular matrix (ECM) of hard tissues favorable for subsequent cell responses and tissue regeneration. During biomineralization, amorphous precursor phases of calcium phosphate infiltrate into preformed collagen fibrils, and upon crystallization, the collagen fibrils are embedded with oriented hydroxyapatite (HA) nanocrystals. We previously demonstrated that hydrogels made of elastin-like recombinamers (ELRs) can template mineralization where minerals were selectively deposited into their frameworks. In this work, we focus on mimicking the nanostructure of the mineralized tissues, namely the intra- and extrafibrillar mineralized collagen fibrils, using the synthetic ELRs. We first electrospun the ELRs into nanofibers and then biomimetically mineralized them via the polymer-induced liquid-precursor (PILP) process. We tested two different ELRs one with a peptide sequence derived from the salivary protein statherin (st-ELR) and a reference one lacking the statherin sequence (ref-ELR). X-ray diffractometry (XRD) and Energy-dispersive X-Ray Spectroscopy (EDS) verified the mineral phase in the ELR nanofibers was HA. Scanning and Transmission Electorn Microscopy (SEM and TEM) analyses revealed that HA nanocrystals were infiltrated into and randomly oriented within the ELR nanofibers. The elastic modulus and hardness of the mineralized ELR nanofibers was increased significantly compared to unmineralized ELR. The mineralized nanofibers promoted the proliferation and osteogenic differentiation of pre-osteoblasts. These results support that a scaffold obtained using this biomimetic strategy and made of mineralized ELR electrospun nanofibers with controlled mineralization and improved mechanical properties has great potential for being used in hard tissue regeneration, such as craniofacial and periodontal and perimplant regeneration as well as tertiary dentin regeneration, as it mimics the structure and mechanical properties of collagen-HA nanocomposites.

## Introduction

Advances in tissue engineering provide new potential in regeneration of damaged hard tissues by combining organic-inorganic hybrid scaffolds with target cells and growth factors. Ideally, the scaffold materials should be biodegradable and eventually be replaced by newly formed tissues, resulting in a transition from artificial materials to native tissues^1^. They should also be designed to mimic natural extracellular matrices (ECMs) which create an in vivo friendly micro-environment for cellular responses^2^. In dentin and bone, the major organic component is type I collagen, which self-assembles in fibrils. Collagen fibrils provide a structural template for mineral deposition so that amorphous calcium phosphate precursors penetrate into the gap zones of the fibrils, crystallize and grow forming hydroxyapatite (HA) nanocrystals oriented parallel to the longitudinal axis of the fibrils^3,4^. The minerals eventually grow exceeding the interstitial spaces within fibrils which results in extrafibrillar mineral deposition. Reproducing the fundamental nanostructure of the extracellular matrix of hard tissues, i.e. the intra- and extrafibrillar mineralized collagen fibrils, has been successfully achieved with various collagen sources via polymer-induced liquid-precursor (PILP) process^5–7^, a biomimetic mineralization system that utilizes poly-aspartic acid as surrogate of non-collagenous proteins (NCPs) to regulate the mineralization of the organic fibrillary matrix. Although polymeric materials have been used to mimic the organic template of natural hard tissues, intrafibrous mineralization of nanostructured synthetic fibrillar templates has been commonly evasive.

The electrospinning technique is a straightforward method to produce non-woven nanostructured fibrous scaffolds that mimic the natural collagen matrix. The electrospun scaffolds have good pore interconnectivity, high surface area and mechanical integrity which are critical for nutrient exchange, cell migration and cell proliferation^8,9^. Various synthetic and natural polymers, such as poly(lactic acid) (PLA)^10^, poly(lactic-co-glycolic acid) (PLGA)^11^, poly(glycolic acid) (PGA)^12^, cellulose^13^, collagen^14^, elastin^15^, chitosan^16^ and their mixtures have been successfully electrospun to obtain fibers. Owing to the advantages in morphologies and mechanical integrity, these electrospun scaffolds can encourage growth of cells and support formation of mineralized tissues^17–19^.

Intra- and extrafibrillar mineralization of collagen fibrils is an important structural feature for providing a physical and chemical environment specifically required for normal osteoblast/odontoblast activity^20–24^. Electrospinning polymer solutions suspended with HA powders have been developed to produce a ceramic-filled fibrous nanocomposite structure that mimic the collagen – HA matrices for potential use as hard tissue substitutes^10,25^. The electrospun nanocomposite fibers; i.e., HA/PLA and HA/polycaprolactone (PCL), significantly enhanced osteoblast attachment and differentiation^10,25^. The presence of HA in PCL/gelatin electrospun scaffolds promoted the adhesion and differentiation of dental pulp stem cells^26^. However, the aforementioned electrospun fibers can only be obtained with a low HA content, namely less than 25 wt%. As the inorganic particles are incorporated in the scaffold as discrete additives, the fibers can become discontinuous and form beads^27^. Electrospun nanofibers have also been used to template mineralization, yet only extrafibrillar mineral deposition was achieved^17,28,29^. The surface of the polymer nanofibers was treated with an alkaline solution or was irradiated to generate hydroxyl or carboxyl groups. During incubation of the activated nanofibers in a simulated body fluid, a layer of bone mineral-like crystals was formed on their surface.

Elastin-like recombinamers (ELRs) have great potential in the regeneration of mineralized tissues as they reproduce the chemistry and structure of the natural polymer and thus, they have good biocompatibility and bioactivity^30,31^. They exhibit a unique inherent thermal response that allows them to undergo phase transition in response to temperature changes^32,33^. Generically, at low temperature the ELRs exhibit random coil conformation in solution. When temperature is raised in the system above the critical reverse transformation temperature (Tf), the polypeptide transform to a β-spiral conformation which leads to self-assembly of the molecules and phase separation of the polymer^34^. Previously, we have demonstrated that coatings made of ELRs containing a statherin-derived peptide (st-ELR) controlled the growth of minerals on nanotopographical surfaces via an enzyme-directed biomimetic mineralization system^35^. Also, using the PILP process we produced scaffolds with HA nanocrystals infiltrated within the frameworks of ELR microporous hydrogels^36^ as well as natural polymer nanofibers (collagen, cellulose, …) with intra and extrafibrillar mineralization^37–39^. Others studies have shown that the st-ELR can guide mineralization of membranes of the polymer with strickingly similar mineral strcutures to the ones in dental enamel^40^, that collagen scaffolds mineralized with the PILP process can recapitulate features of the bone cellular and extracellular microenvironment, including its protein-guided biomineralization, nanostructure, vasculature, and innervation^41^ and that the mineralized collagen scaffolds via PILP could be combined with antimicrobial peptide nanofibers to impart anti-infection properties to bone-regenerative scaffolds^42^. Altogether showing the biomimicry potential and versatility of the combination of ELRs and mineralization using a non-classical biomineralization process such as the PILP.

In this study, we fabricated non-woven nano-fibrous ELR scaffolds using the electrospinning technique and biomimetically mineralized them via the PILP process. We hypothesized that the electrospun ELR nanofibers can template mineralization to obtain an intrafibrous mineralized structure. With this controlled mineralization, the mineralized ELR scaffold will mimic relevant structural features and mechanical properties of dentin/bone tissues. Therefore, the mineralized ELR scaffold will have the potential to promote bioactive hard tissue-related cell responses for enabling and favoring tissue regeneration.

## Experimental section

### Materials

Two recombinant polymers, st-ELR (71.5 kDa) and ref-ELR (51.9 kDa), were purchased from Technical Proteins Nanobiotechnology (Valladolid, Spain). They were synthesized using genetic engineering techniques, as described previously^43^. Table 1 shows that the ref-ELR, control polymer, contains the repeating pentapeptide sequences, VPGIG and VPGKG, derived from the natural tropoelastin amino acid sequence. st-ELR is an amphiphilic triblock polymer that additionally includes a highly acidic sequence, SN_A_15 (DDDEEKFLRRIGRFG), derived from the salivary protein statherin with an active role in mineral regulation in the oral environment^44^. Poly-*L*-aspartic acid (Mw 27 kDa) was purchased from Alamanda Polymers (Huntsville, Al, USA). Fetal bovine serum (FBS), α-modified medium (α-MEM), penicillin/streptomycin (P/S), and trypsin-EDTA were purchased from Invitrogen (Grand Island, NY, USA). ALP protease inhibitor was purchased from Thermo Scientific (Rockford, IL, USA). Bio-Rad DCTM protein assay kit was purchased from Bio-Rad (Hercules, CA, USA). Mouse osteocalcin Elisa Kit was purchased from Biomedical Technologies (Stoughton, MA, USA). All other chemicals were purchased from Sigma-Aldrich (St. Louis, MO, USA).

**Table 1:** Primary peptide sequence of st-ELR and ref-ELR.

Name	Sequence
st-ELR	MESLLP[[(VPGIG)_2_VPGKG(VPGIG)_2_]_2_DDDEEKFLRRIGRFG[(VPGIG)_2_VPGKG(VPGIG)_2_]_2_]_3_(VPAVG)_20_[[(VPGIG)_2_VPGKG(VPGIG)_2_]_2_DDDEEKFLRRIGRFG[(VPGIG)_2_VPGKG(VPGIG)_2_]_2_]_3_V
ref-ELR	MESLLP[(VPGIG)(VPGIG)(VPGKG)(VPGIG)(VPGIG)]_24_

### Fabrication of electrospun ELR nanofibers

st-ELR and ref-ELR were added in 0.6% NaCl aqueous solution at concentrations of 25 and 30 w/v %, respectively. They were stored at 4 °C overnight to dissolve completely and then loaded in a 1-mL syringe connected to a blunt-ended 18 gauge needle. A voltage difference of 18 kV was applied between the needle and the target and the collection distance was set at 15 cm A solution flow rate of 3 µl/min was sustained during electrospinning in all experiments. Polished titanium discs with 4 mm in diameter were served as collecting substrate in order to obtain same surface area of specimens used for the cell culture study. A thin layer of ELR nanofibers about 4 μm-thick were deposited on these discs at room temperature. After air-drying overnight at room temperature, the ELR nanofibers on the discs were cross-linked with hexamethylene diisocyanate (HMDI) in acetone (10 v/v %) overnight to improve their physical stability as they would otherwise dissolve in the PILP solution. The cross-linked ELR nanofibers were then washed thoroughly with acetone and then deionized water. They were lyophilized before material characterization and mineralization.

### Biomimetic mineralization via the PILP process

The cross-linked samples were mineralized by the PILP process following the protocol described previously^45,46^. Briefly, solutions of 9 mM CaCl_2_ and 4.2 mM K_2_HPO_4_ were prepared in Tris-buffered saline (TBS, pH = 7.4 at 37 °C) separately. Poly-*L*-aspartic acid (PASP, MW = 27 kDa) was added in Ca-solution at the concentration of 100 µg/mL. Then, equal volumes of these two solutions were mixed together gently to make the final concentration of Ca, P and PASP at 4.5mM, 2.1mM and 50 µg/mL, respectively. st-ELR and ref-ELR samples were immersed into the mineralization solution under vacuum for 30 min to remove air bubbles within the scaffolds. After degassing, the mineralization was conducted in an oven at 37 °C up to 7 days in closed vials. The PILP solution was refreshed every 3 days. After mineralization, the samples were washed with deionized water for three times consecutively and lyophilized.

### Characterization

#### Scanning Electron Microscopy (SEM)

SEM (JEOL6500, Japan) was used to analyze surface morphologies of st-ELR and ref-ELR fibers before and after mineralization. Prior to SEM analysis, all the specimens were coated with a thin layer of platinum. All the images were recorded at an accelerating voltage of 5 kV. Energy Dispersive Spectrometry (EDS) was conducted at 15 kV to evaluate chemical composition of the mineralized fibers. Some SEM images were analyzed by ImageJ, available as free software from the National Institutes of Health (http://imagej.nih.gov/ij/), to determine the diameter of the fibers at predetermined mineralization times. The percentage of increase in fiber diameter (IFD%) was calculated using the following equation:

$$\:IFD\%=\raisebox{1ex}{$(D-{D}_{0})$}\!\left/\:\!\raisebox{-1ex}{${D}_{0}$}\right.\:\times\:100\%$$

Where D is the average diameter of electrospun fibers at the predetermined mineralization time, D_0_ is the average diameter of electrospun fibers before starting the mineralization process.

#### Transmission electron microscopy (TEM)

TEM (Tecnai T12, FEI, USA) was used to analyze the crystal structure and orientation of the minerals in the electrospun fibers at an accelerating voltage of 120 kV. The mineralized st-ELR and ref-ELR fibers were embedded into epoxy resin and cured at 60 °C overnight. They were then cut by an ultramicrotome (Leica Reichert UltraCut S) at room temperature and collected on 300-mesh copper grids. Both the longitudinal and transverse sections of the mineralized fibers were evaluated. Selected area electron diffraction (SAED) was conducted to assess the crystallinity and orientation of the deposited minerals.

#### X-Ray diffractometry (XRD)

The crystalline phases of minerals were also determined by XRD (D8 Discover, Bruker AXS). Spectra of st-ELR and ref-ELR fibers at the different mineralization time points, as well as of a bovine dentin slab, were collected at 40 kV and 40 mA with 2θ ranging from 20° to 50°. The results were analyzed using the JADE8 software (Materials Data Inc., Livermore, CA, USA).

#### Nano-indentation

Nano-indentation (Hysitron Triboindenter, Minneapolis, USA), was used to evaluate the mechanical properties of st-ELR nanofibers after 7 days of mineralization. Polished bovine dentin was used as control sample. A Berkovich tip with a radius of 150 nm was selected as an indenter. Optical imaging of the sample using a microscope allowed precise control between the sample position and the indenter to ensure that results from each test were obtained solely from the individual fiber. Each indentation used a trapezoidal load function consisting of a linear loading segment of 10 s followed by a 5-second holding at a predetermined peak force (50–2000 µN) and a linear unloading segment of 10 s. Twenty indents on each sample were performed. Reduced elastic modulus (Er) and hardness (H) were determined using the compliance method of Oliver-Pharr^47^.

### Cellular response

#### Cell culture

Mouse calvarial preosteoblasts (MC3T3-E1; ATCC, Manassas, VA, USA) were cultured in α-MEM, supplemented with 10% FBS, and 1% of penicillin/streptomycin. They were cultured in an incubator at 37 °C with 5% CO_2_ and passaged 3 times before being seeded on the ELRs samples.

#### Cell proliferation

Cells, at the density of 2000 cells/well, were seeded on mineralized and un-mineralized st-ELR and ref-ELR fibers that were deposited on titanium discs. 2-mm thick bovine dentin slabs from bovine incisor were used as control. Prior to cell seeding, all the samples were soaked in 70% ethanol for 40 min and then exposed to UV light for another 40 min for disinfection. The samples were then washed thoroughly with Hank’s buffered salt solution (HBSS) and placed in a 48-well plate before cell seeding. Cell culture medium was refreshed every three days. After 1, 3 and 6 days of culture, cells were fixed with 4% paraformaldehyde (PFA) for 20 min in ice bath, and then stained with 2 µg/mL DAPI for 10 min. Immunofluorescence Microscopy (Eclipse E800, Nikon, Japan) was used to determine cell number per field by counting the stained nucleus in images taken at 20X magnification.

#### Cell differentiation

Prior to cell seeding, all the samples were disinfected using the aforementioned protocol and then transferred to a 96-well plate. MC3T3-E1 cells were seeded on the scaffolds at the density of 10,000 cells/well and cultured with 300 µl α-MEM. After 2 days of culture, cells were then reached confluence and were cultured with osteogenic differentiation medium (α-MEM with 10% FBS, 1% P/S, 50 µg/mL ascorbic acid and 0.01M *β*-glycerophosphate). During cell differentiation, half the volume of the medium was refreshed every 3 days. The differentiation biomarkers, alkaline phosphatase (ALP) activity and osteocalcin production (OCN) were evaluated at 7, 14 and 21 days of culture. To test ALP activity, the cultured samples were washed 3 times with PBS and immersed in 300µL lysis buffer containing 1% Triton X-100, 0.1mM MgCl_2_, and 0.15 M Tris-base at pH 10.5. After 10 min of incubation at room temperature, the cell lysates were collected and centrifuged at 3000 rpm for 10 min. ALP activity was tested using ALP assay kit. Briefly, AMP reaction buffer was prepared by dissolving one 20 mg tablet of p-nitrophenylphosphate in 5 ml deionized water followed by addition of 11.25 µl 1:4 diluted AMP and 6µL 2 M MgCl_2_. 100µL of AMP buffer was added in a 96 well plate followed by the addition of 2µL of cell lysates from each sample. The mixture was incubated in the oven at 37 °C for 2 h, and then subjected to synergy TM 2 multi-mode microplate reader (BioTek, US) to measure the absorbance at 410 nm. The medium was collected after 7, 14 and 21 days of cell culture protein production of osteocalcin was measured using the Osteocalcium Elisa kit following the protocol provided by the manufacturer^35^. The data of ALP activity and OCN levels was normalized using the total protein content, which was obtained using Bio-Rad ^DCTM^ protein assay kit (BioRad, Hercules, CA) following the manufacturer’s protocol. All cell experiments were performed using two independent biological replicates. For each biological replicate, four technical replicates (samples) were analysed for each experimental group.

#### Statistical analysis

Statistical analysis was performed by first assessing the normality of the data distribution using the Kolmogorov–Smirnov test, which allowed the application of parametric statistical methods. Homogeneity of variances was subsequently evaluated using Levene’s test. Statistical significance between groups (*p* < 0.05) was then assessed using one-way ANOVA followed by Tukey’s multiple comparisons test.

## Results

### Electrospinning and mineralization of ELR fibers

**Fig. 1:**
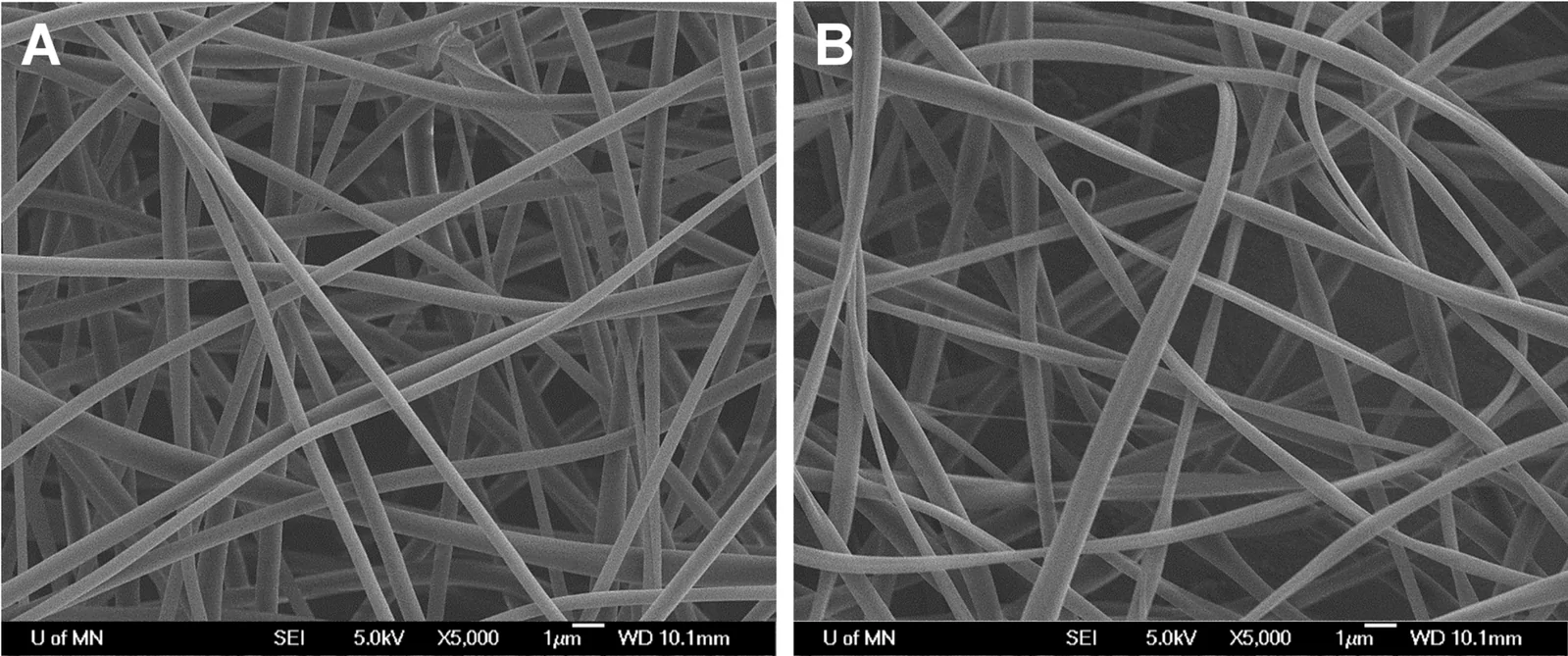
SEM images of electrospun nanofibers of st-ELR (**A**) and ref-ELR (**B**).

Using the electrospinning technique we obtained ELR fibers with diameters of several hundreds of nanometers. Figure 1 shows that both st-ELR and ref-ELR nanofibers exhibited a loose non-woven structure. The diameter of the st-ELR nanofibers (406 ± 74 nm) was smaller than that of the ref-ELR nanofibers (741 ± 257) (Table 2). Some of the ref-ELR fibers showed a ribbon-like shape.

**Fig. 2:**
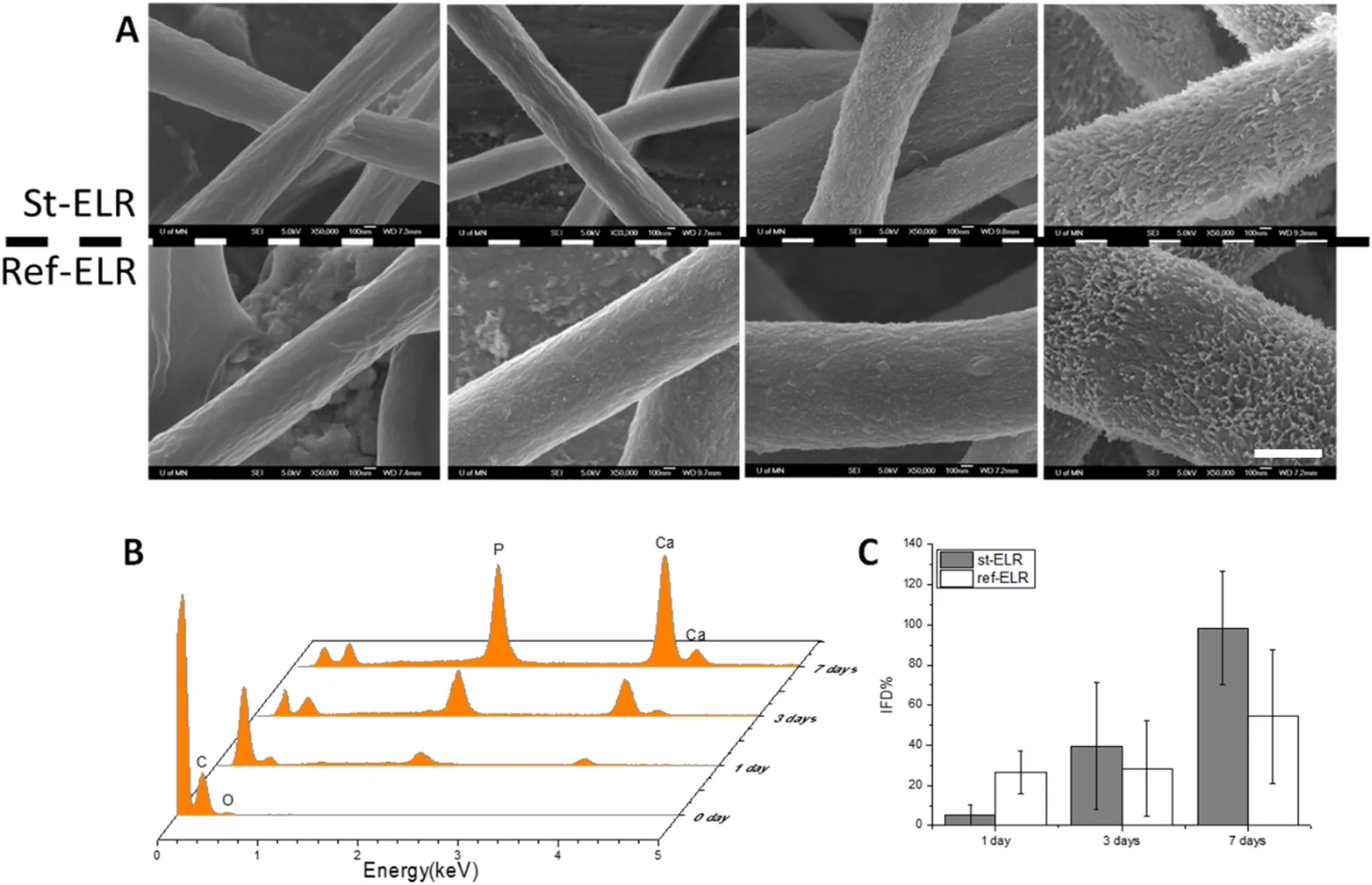
(**A**) SEM images of st-ELR (1st row) and ref-ELR (2nd row) nanofibers after 0, 1, 3 and 7 days of mineralization. (**B**) EDS spectra of the mineralized st-ELR electrospun nanofibers and (**C**) percentage of increase in fiber diameter at different periods of mineralization calculated by analysis of all fibers included in *n* = 3 images of each sample. Bar is 500 nm.

Before mineralization, the surface of ELR nanofibers appeared smooth with small striations (Fig. 2A). A remarkable change in surface morphology was observed as the mineralization proceeded. After 3 days of mineralization the surface of the nanofibers had a rugged uneven appearance along the whole length of the fiber. A continuous layer of needle-like minerals was observed on the surface of the ELR fibers after 7 days of mineralization. The diameters of the fiber were continuously increased while the mineralization process progressed. The diameter of st-ELR and ref-ELR reached 805 ± 114 nm and 1143 ± 394 nm after 7 days of mineralization, respectively (Table 2); therefore, the %IFD was significantly higher for st-ELR than ref-ELR nanofibers after 7 days of mineralization (D7) (Fig. 2C).

**Table 2:** Ca/P ratio of the minerals deposited in ELR nanofibers determined by EDS and the diameter of st-ELR and ref-ELR nanofibers measured from the SEM images. N/A: not available. Values are mean ± standard deviation for *n* = 3 fields.

Day	Ca/*P* ratio		Diameter (nm)	
	st-ELR	ref-ELR	st-ELR	ref-ELR
0	N/A	N/A	406 ± 74	741 ± 257
1	1.35 ± 0.03	1.34 ± 0.07	417 ± 22	919 ± 174
3	1.39 ± 0.10	1.45 ± 0.03	567 ± 128	951 ± 176
7	1.59 ± 0.11	1.59 ± 0.08	805 ± 114	1143 ± 394

EDS was performed during the SEM analysis to determine the chemical composition of the obtained minerals. Calcium and phosphorus were detected after 1 day of mineralization. The intensity of both Ca and P signals increased gradually with mineralization time (Fig. 2B). After 1 day of mineralization, the molar Ca/P ratio was 1.35 and 1.34 in st-ELR and ref-ELR, respectively. At day 7 of mineralization both mineralized ELRs had Ca/P ratio that increased to 1.59, which is a value comparable to the one for natural dentin (Ca/*P* = 1.61)^48^.

### Crystallographic structure

**Fig. 3:**
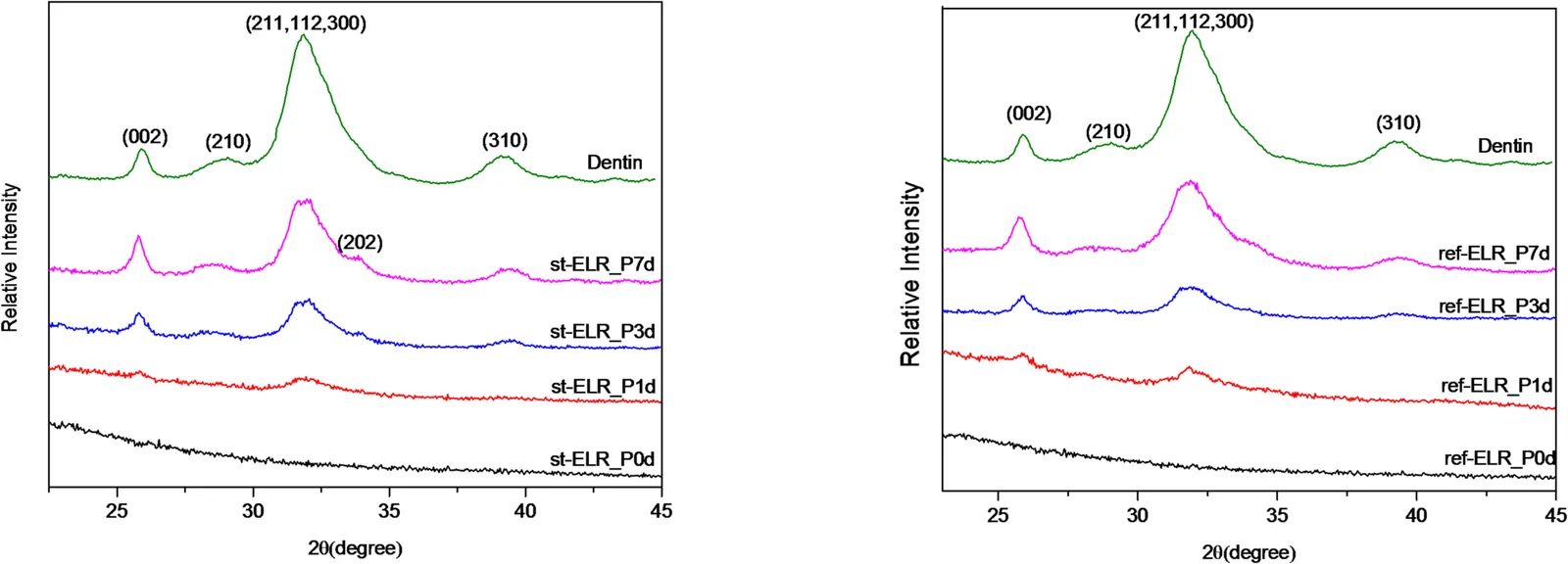
XRD of electrospun st-ELR (**A**) and ref-ELR (**B**) nanofibers after 0, 1, 3 and 7 days of biomimetic mineralization. The spectrum of bovine dentin was used for comparison. Peaks were assigned to HA crystals according to reference JCPDS card No. 9–432.

X-ray microdiffraction was used to analyze the crystallinity of the mineral deposited in ELR nanofibers. The presence of HA nanocrystals in st-ELR and ref-ELR nanofibers over time of mineralization is shown in Fig. 3. The main characteristic peaks for HA appeared after 1 day of the PILP treatment which were at 2θ of 26° and 32° corresponding to (002) planes and overlapping (211), (112) and (300) planes, respectively. These peaks progressively increased in intensity over the mineralization time. The formation of HA was confirmed from the spectra of both st-ELR and ref-ELR. After 7 days of mineralization, the XRD pattern for st-ELR and ref-ELR was very similar to that of dentin.

### Crystal distribution and orientation

**Fig. 4:**
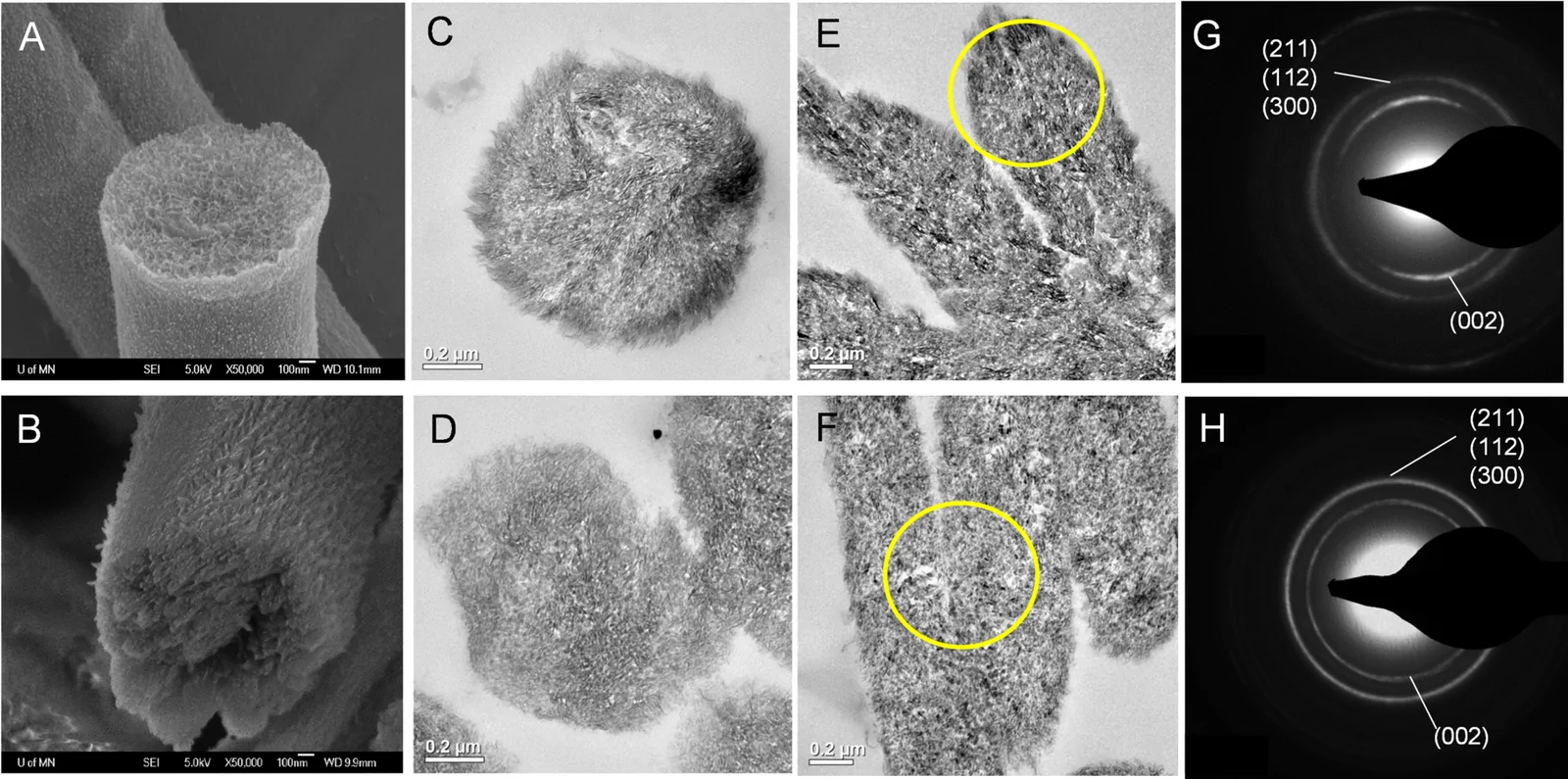
SEM and TEM analysis of ELR nanofibers after 7 days of mineralization. SEM images of the fractured surfaces of st-ELR (**A**) and ref-ELR (**B**) nanofibers. TEM images of the transverse-sections of mineralized st-ELR (**C**) and ref-ELR (**D**) nanofibers. (**E**) and (G) TEM image of the mineralized st-ELR nanofibers and the corresponding SAED pattern generated from the region circled in (E). (**F**) and (**H**) TEM image of the mineralized ref-ELR nanofibers and the corresponding SAED pattern obtained from the region circled in (F).

We performed SEM analysis on the fracture surface of the mineralized fibers to assess the infiltration of the minerals in the interior of the ELR nanofibers (Fig. 4A and B). The fractured surface of the inner side of the nanofibers had similar morphology as the one for the exterior surface of the fibers, indicating the occurrence of intra-fibrous mineralization. TEM results, from both longitudinal and transverse sections of the mineralized fibers, further confirmed that homogenous intra- and extrafibrillar mineralization was obtained in both st-ELR and ref-ELR fibers (Fig. 4C-F). In most cases, the crystals were randomly oriented in the fibers; however, alignment of HA nanocrystals along some of the st-ELR fiber axis were observed and verified by the arcing of the (002) planes in the corresponding SAED pattern (Fig. 4G).

### Mechanical properties

Table 3 shows reduced moduli of elasticity and hardness values for mineralized st-ELR samples and dentin in dry and hydrated conditions that were determined from the load-displacement curves at a depth of 500 nm. The mechanical properties of st-ELR fibers before mineralization were too low to be measured by nanoindentation. However, mineralization led to a significant increase of elastic modulus and hardness of st-ELR. The elastic modulus of the mineralized st-ELR fibers, in dry and wet states, was 0.99 ± 0.77 GPa and 0.17 ± 0.20 GPa, respectively and the corresponding hardness value were 0.058 ± 0.043 GPa and 0.005 ± 0.005 GPa. These values are much lower than those of dentin which had elastic modulus of 22.7 ± 2.7 (dry) and 10.7 ± 4.2 (wet) GPa, and hardness value of 1.01 ± 0.23(dry) and 0.28 ± 0.17 GPa (wet). The values of mineralized st-ELR were also lower than that of bovine cortical bone^6^, but they are comparable to the ones reported for rehydrated mineralized collagen film which had elastic modulus of 0.177 ± 0.031 GPa and hardness value of 0.008 ± 0.003 GPa in wet state^6^.

**Table 3:** Mechanical properties, reduced modulus of elasticity (Er) and hardness (H), of the electrospun st-ELR fibers after 7 days of biomimetic mineralization and control samples of bovine dentin (*n* = 20 indents per sample).

	Reduced Modulus (Er) [GPa]		Hardness (H) [GPa]	
	dry	wet	dry	wet
st-ELR_7D	0.99 ± 0.77	0.17 ± 0.20	0.058 ± 0.043	0.005 ± 0.005
Dentin	22.7 ± 2.7	10.7 ± 4.2	1.01 ± 0.23	0.28 ± 0.17

### Pre-osteoblast proliferation and differentiation

**Fig. 5:**
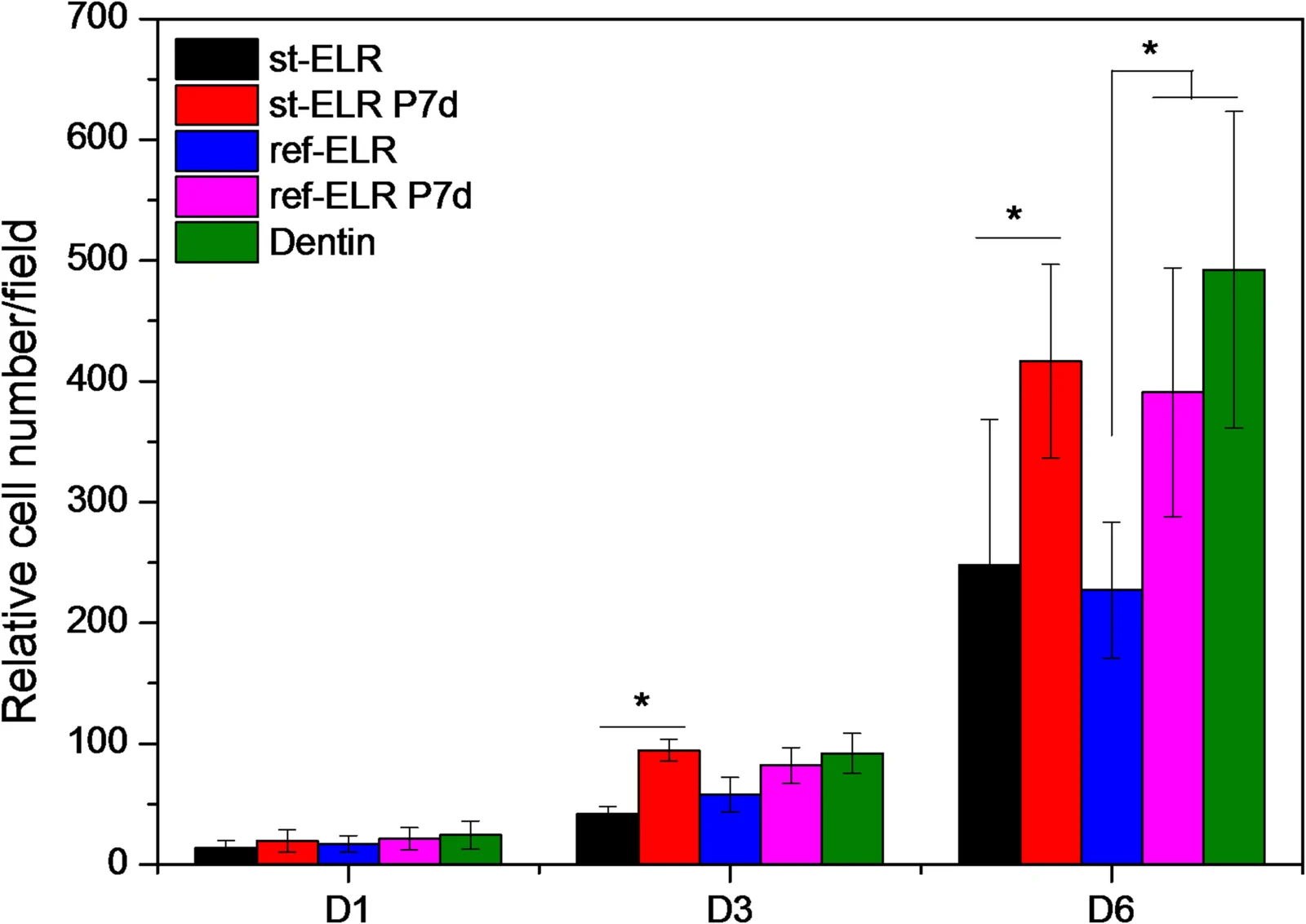
Cell proliferation of MC3T3-E1 cells after 1 (D1), 3 (D3) and 6 (D6) days of culture on control dentin slabs, unmineralized ELRs (st-ELR, ref-ELR), and ELRs after 7 days of biomineralization (st-ELR_P7d, ref-ELR_P7d) (*n* = 4 for 2 biological replicates).

Proliferation and differentiation of mouse calvarial pre-osteoblastic cells (MC3T3-E1) was determined when cells were cultured in vitro on unmineralized and mineralized ELRs samples. After 1 day of culture, no significant differences in cell proliferation were observed among all groups tested and control dentin samples (Fig. 5). After 3 days of culture, mineralized ELRs and dentin slabs showed higher cell proliferation numbers than the ones for unmineralized st-ELR nanofibers(*p* < 0.05) (Fig. 5). Significant increase in cell number after 7 days was observed in mineralized ELRs and dentin when compared to the unmineralized ELRs (*p* < 0.05, *N* = 4) (Fig. 5).

**Fig. 6:**
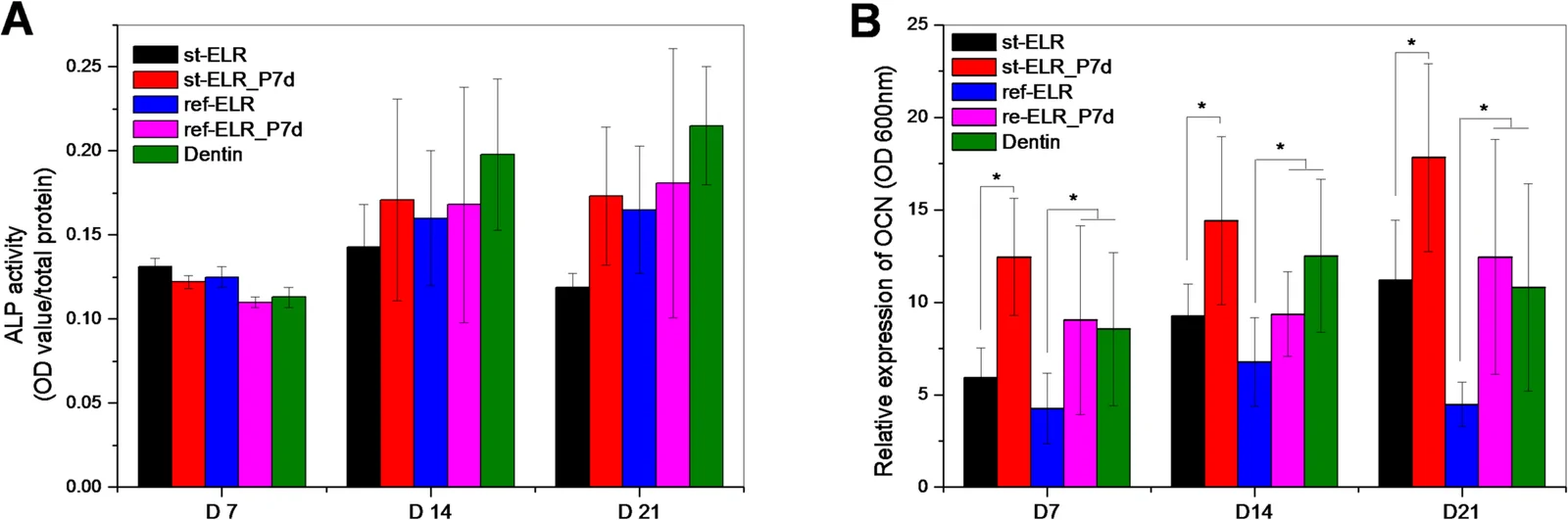
In vitro steogenic differentiation; (**A**) ALP activity and (**B**) Osteocalcin production of MC3T3-E1 cells after 7 (D7), 14 (D14) and 21 (D21) days of culture on control dentin slabs, unmineralized ELRs (st-ELR, ref-ELR), and ELRs after 7 days of biomineralization (st-ELR_P7d, ref-ELR_P7d) (*n* = 4 for 2 biological replicates).

Although cells showed a continuous increase in ALP activity over the 21 days of culture, except for unmineralized st-ELR samples, no significant differences in cellular ALP activity were determined between any of the tested groups at any of the time points where cells were analyzed (Fig. 6A). However, cells cultured on mineralized ELRs and dentin showed a significantly higher production of OCN than unmineralized ELRs for all periods of culture (Fig. 6B).

## Discussion

Significant scientific and technological advances have been made to enable the use of electrospinning to produce scaffolds for hard tissue regeneration. The incorporation of inorganic nanocomponents within the polymeric nanofibers that improve the mechanical and biological properties of the scaffold is the main focus of investigation and development. Instead of electrospinning composite solutions or nucleating and growing minerals on the surface of the fibers, biomimetic intrafibrous mineralization of the electrospun polymeric nanofibers is a promising approach for the development of artificial hard tissue matrices. This process mimics the one by which mineralization of natural collagen fibrils occurs and thus, it better recapitulates the structure of native hard tissues. In this study, we demonstrated that electrospun ELR nanofibers can be intra- and extrafibrillar ly mineralized using the PILP process. The mineral, HA nanocrystals, was homogeneously distributed and thoroughly infiltrated in the nanofibers without disrupting the original fiber morphology. As a result, the mineralized ELR nanofibers improved proliferation and osteogenic differentiation of MC3T3-E1 cells, which indicates that these scaffolds might be used for successful guidance of hard-tissue regeneration.

In the pursuit of developing synthetic templates that can induce mineralization with controlled morphology that mimics natural hard tissues, electrospun nanofibers were used to template mineral growth, yet only extrafibrillar mineralization was achieved^17,29^. However, during natural biomineralization of dentin and bone both extra- and intra-fibrillar mineralization of the collagen matrix takes place. Intrafibrillar mineralization is critical for controlling the mineral orientation and its spatial distribution in the fibrillar matrix and determining its mechanical properties. Previous work has shown that the PILP process is capable of reproducing the nanostructure of hard tissues, the mineralized collagen fibrils^7,49^. In the electrospun ELR nanofibers, the biomimetic mineralization starts with intra-fibrous mineral deposition and is followed by the extrafibrous growth of crystals. The diameter of the electrospun fibers significantly increased with biomineralization time. Strong signals of Ca and P were detected after 3 days of mineralization, suggesting the presence of the crystals in the ELRs scaffolds. However, ELR fibers after 3 days of mineralization did not show a marked change in surface morphology. It was only after 7 days of mineralization that the surface of the ELR fibers were decorated with needle-like minerals. This suggests the initial penetration of the minerals in the fibers that eventually get thoroughly infiltrated by crystals and the minerals finally erupt through the fiber surface. In organic matrix-templates, mineralization takes place within defined and restricted compartments or spaces. The organic matrix should contain pores or channels to serve as sites for mineral infiltration and deposition^50^. At the same time, the mineral should be stabilized in solution as an amorphous precursor that can penetrate into the organic matrix before crystallization^51,52^. In bone, non-collagenous proteins stabilize the amorphous calcium phosphate phase that is able to infiltrate into the interstitial spaces of individual collagen fibrils^51–53^. ELRs exhibit microphase separation in response to the increase of temperature as a result of their reverse transition^54^. In the electrospun ELR fibers, as in our previous work with ELR hydrogels^36^, the phase transition might result in formation of nanopores to facilitate the infiltration of the minerals. However, it should be noted that here the spatial distribution of HA nanocrystals in the ELR fibers was not controlled. Biomineralization in an organic matrix is a confinement controlled process^55^, orientation of HA crystals is regulated by the arrangement of the polymeric chains in the organic matrix. Collagen fibrils have a characteristic D-periodic banding pattern of 64–67 nm^56,57^. The mineralization starts at the gap zones of the collagen fibrils and then the mineral grows into the overlap zones so the *c*-axis of HA nanocrystals is aligned with the fibril axis^52^. In contrast to the natural collagen structures, we only detected at the ends of the ELRs fibers preferentially aligned HA nanocrystals with their [0 0 1] roughly parallel along the fiber axis (Fig. 4E). Most HA nanocrystals were randomly oriented in the ELR fibers as, most likely, the mineral infiltrated the fiber from any point of their periphery (Fig. 4F).

Mineralization rate depends on the capability of infiltration of the mineral nanoclusters into the organic matrix. st-ELR contains a peptide, SN_a_15, which is a synthetic analog of the N-terminal 15 residue sequence of human salivary statherin^44^. This peptide has high affinity for calcium phosphate minerals^44^ and thus, we hypothesized that its presence in the ELR sequence attracts minerals to the fibers, thereby increasing the mineralization rate. ref-ELR exhibited higher solubility in aqueous solution and lower viscosity than that of the triblock copolymer st-ELR, which we attributed to the lower molecular weight (51.9 kDa vs. 71.5 kDa) and less proportion of polar residues of ref-ELR compared to ref-ELR (Table 1). In order to electrospin ref-ELR nanofibers, we increased the concentration of ref-ELR to 30 w/v %, yet the viscosity was still lower than that of the st-ELR (25 w/v %). This low viscosity and low molecular weight influenced the outcome of electrospinning, producing ref-ELR fibers with larger and more disperse diameters than the st-ELR fibers (Fig. 2C). Besides, it is well known that polymers with high molecular weight while being electrospun tend to orient their chains along the fiber axis as they have extensive entanglements and long relaxation time^58^. Given the difference in molecular weight and viscosity, one can expect that st-ELR fibers had more parallel molecular arrangements along the fiber axis than in the case of ref-ELR fibers. The parallel array of the molecules in st-ELR fibers might also contribute to the high percentage of increased fiber diameter during mineralization, pertinent to the high degree of mineralization.

Biomimetic mineralization enhanced the mechanical properties of st-ELR. Although the elastic modulus and hardness of mineralized st-ELR were lower than that of bovine dentin and cortical bone (Table 3), the values were comparable to the mineralized crosslinked collagen in wet state. The lower mechanical strength of st-ELR is presumably due the shorter mineralization time and the less compacted structure than the native mineralized matrices. st-ELR fibers were mineralized for only 7 days while the collagen films in our previous study was mineralized for 14 days^6^. However, the mechanical strength of mineralized st-ELR is rigid enough for supporting osteoblast cell activity, as matrix with elasticity at about 0.1 GPa is demonstrated to be osteoinductive^59^.

Cell fate and functions are regulated by chemical and physical cues^45^. Surfaces with micro/nano topography and high stiffness^45,59,60^ favor appropriate responses by mineralized tissue-related cells, such as primary osteoblast and odontoblast-like cells. Scaffolds with a nano-fibrous structure and high stiffness promote the osteogenic and odontogenic cell growth and differentiation^26,61^. In this study, surface chemistry, stiffness, and substrate properties were not systematically controlled, which limited our ability to directly attribute the observed cellular responses specifically to the effects of mineralization. Notwithstanding, the fibrous structure and mineralization of ELR fibers might have led to an enhancement in cell proliferation and differentiation as the mineralized ELR fibers mimicked the mineralized collagen fibrils, the primary component of bone and dentin. Intra- and extrafibrillar mineralization resulted in significant improvement of mechanical properties (Table 3). Such fibrous structure, coupled with mechanical cues, created a favorable micro-environment for cell response. The cellular OCN production on the mineralized samples was higher after 14 and 21 days of culture than on the unmineralized samples (Fig. 6B). In fact, previous studies have shown that both non-mineralized ELRs and mineralized ELRs with different structural features and morphologies can enhance bone regeneration in vitro and in vivo^36,39,40,62,63^. However, the biomimetic fibrillar architecture and extensive mineralization achieved in the ELR materials investigated in our work represent a distinct and more physiologically relevant approach, designed to more closely reproduce key structural and compositional features of native mineralized extracellular matrices.

## Conclusions

The potential of elastin-like recombinamers to control calcium phosphate mineralization in a manner that mimics biomineralized collagen in natural calcified tissues has been investigated. We demonstrated that electrospun st-ELR and ref-ELR nanofibers induced both intra- and extrafibrillar mineralization through the PILP process, leading to enhanced proliferation and differentiation of primary osteoblast cells. These findings indicate that mineralized electrospun nanofibrous ELR scaffolds may provide an optimized biomimetic physical and chemical environment capable of stimulating cellular activity. This work therefore presents a promising strategy for the development of biomimetic scaffolds potentially suitable for hard tissue regeneration.

## Data Availability

The authors declare that the data supporting the findings of this study are available within the paper . Should any raw data files be needed in another format they are available from the corresponding author upon reasonable request.
